# Experiences of Nurse Practitioners in Communicating Bad News to Cancer Patients

**DOI:** 10.6004/jadpro.2016.7.5.2

**Published:** 2016-07-01

**Authors:** Virginia Ruth Corey, Priscilla Gage Gwyn

**Affiliations:** 1 Marian University at Saint Thomas Health, Nashville, Tennessee;; 2 NeuroOncology Center, Florida Hospital, Orlando, Florida

## ABSTRACT

How oncology practitioners communicate with patients has a strong impact on quality health care. Good communication facilitates positive experiences for both practitioners and patients alike, yet many practitioners report they are inadequately prepared for delivering bad news to cancer patients and often have negative experiences due to poor communication. Using a qualitative exploratory descriptive design this study sought to understand the experiences of nurse practitioners (NPs) when communicating bad news to cancer patients. Methodology consisted of two steps. First, five Florida-licensed NPs with at least 2 years of oncology experience were educated on the use of the SPIKES protocol and utilized it in clinical practice for 30 days. Second, semistructured individual interviews were conducted to record their perceptions of using the SPIKES protocol. Thematic analysis results support the concept that "the experiences of the nurse practitioner when delivering bad news to cancer patients are shaped by their own communication skills." Educating oncology NPs in using the SPIKES protocol when delivering bad news has the potential to positively impact the experiences of both NPs and patients.

## ARTICLE

In 1995, Breaking Bad News Consensus Guidelines were published (Girgis & Sanson-Fisher, 1995). They called for improved medical provider communication with patients outlining there was a lack of research in the specific steps in delivering bad news and recommending research be undertaken both on patient and provider perceptions in the delivery of such news.

The SPIKES protocol is one such step-wise framework for delivering bad news that can support discussions, improve communication, enhance practitioner confidence, support patient-centered care, and facilitate a shared decision-making model in patient care. This tool is an acronym for the steps in which to deliver bad news (see Table 1). Since the 1990s, research has continued to expand on communication tool training and implementation for medical providers and students in training to be health-care providers.

**Table 1:**
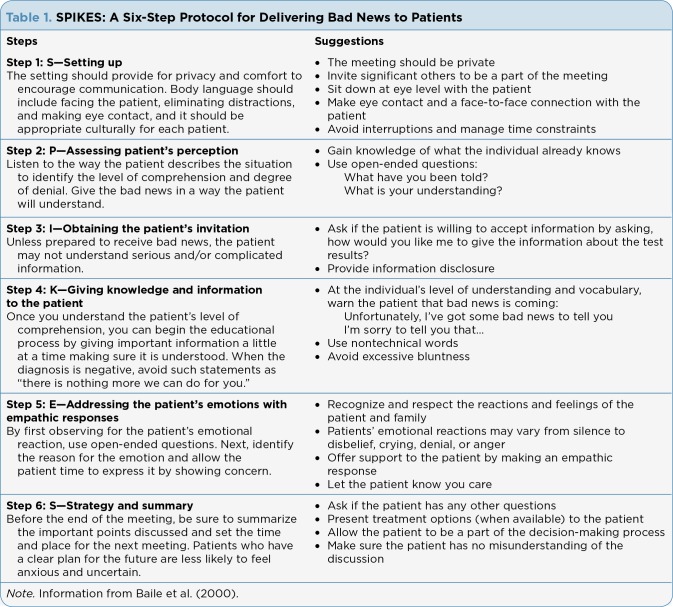
SPIKES: A Six-Step Protocol for Delivering Bad News to Patients

## REVIEW OF THE LITERATURE

Research has shown that when poor communication is used to deliver bad news, cancer patients often suffer significant emotional and psychological trauma and are at risk of receiving less-than-optimal quality of care (Horne, Seymour, & Payne, 2011; Thorne et al., 2013). Research demonstrates that communication skills training and using a tool to assist in the development of a therapeutic relationship positively impact patients and their relatives (Epstein & Street, 2007; Fujimori et al., 2014; Merckaert et al., 2013; Morgans & Schapira, 2015).

More recently, research in this important area has expanded to evaluate the way in which formal communication skills training impacts not only patients, but also the "deliverer" of the bad news, such as the medical student (Kiluk, Dessureault, & Quinn, 2012; Skye, Wagenschutz, Steiger, & Kumagai, 2014); nursing student (Little & Bolick, 2014); nurse (Baer & Weinstein, 2013; Banerjee et al., 2016; Bylund et al., 2011); clinical nurse specialist (Mishelmovich, Arber, & Odelius, 2016); physician (Bylund et al., 2011; Fujimori et al., 2014; Grainger, Hegarty, Schofield, White, & Jefford, 2010; Merckaert et al., 2013); or other related nonphysician health-care providers such as the social worker, nurse practitioner (NP), or physician assistant (PA; Bylund et al., 2011; Parker et al., 2010).

However, although research on the "deliverer" is beginning to be amassed, it appears that few studies have examined nurse practitioners as a separate entity (Rosenzweig, 2012), and for the few that included NPs in research as subjects, they were often grouped with nurses (Bylund et al., 2011). This lack of research with advanced practitioners demonstrates the need for empirical research with NPs and PAs, as these health-care providers are on the front lines of delivering bad news and need expert communication skills.

Historically, physicians may have uncertainty in deciding the best way in which to share difficult news with patients (Curtis et al., 2002; Park et al., 2010). Like their physician colleagues, many NPs report they are inadequately prepared or trained for this task and often have negative experiences (Rosenzweig, 2012; Warnock, Tod, Foster, & Soreny, 2010). Baile et al. (1997, 1999) found that less than 10% of providers were trained to deliver bad news in a manner that supported the needs of cancer patients, leading to their experiencing significant suffering as recipients of poor communication (National Cancer Institute [NCI], 2015; Thorne et al., 2013).

To reiterate, research with medical students and physicians demonstrates that the skill of communicating bad news can be taught through communication skills training. Therefore, formal communication skills training is beginning to be standardized in medical provider curricula but has not traditionally been a routine part of NP education. However, many NPs currently in practice have often learned by observing physicians, who may also have limited communication skills training themselves before this was standard in their medical education (Rosenzweig, Clifton, & Arnold, 2007).

Literature review reveals that implementing education may not be enough to enhance the feeling of confidence with the skill and that continued practice in the clinical setting is warranted, as it can be more challenging in the clinical setting than in practice (Barth & Lannen, 2011; Little & Bolick, 2014). Further research with additional communication training for NPs in clinical practice is needed.

To meet the communication needs of patients, NPs must learn to communicate in a manner that delivers bad news in a supportive fashion while at the same time allowing patients to be a vital part of their cancer treatment plan (Baile et al., 2000), a concept supported in the theory put forth by the National Cancer Institute’s Conceptual Framework for patient-centered communication in cancer care, as adapted by the authors (Figure; Epstein & Street, 2007; Perocchia et al., 2011). Delivery of bad news using frameworks such as the SPIKES protocol can help facilitate these vital elements in this theory (Kaplan, 2010; Kiluk et al., 2012; Little & Bolick, 2014; Morgans & Schapira, 2015).

**Figure:**
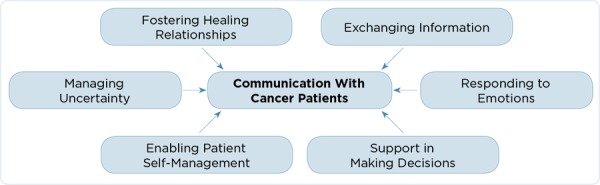
Visual representation of the National Cancer Institute’s Conceptual Framework for patientcentered communication in cancer care and caring through communication with cancer patients. Adapted from Epstein & Street (2007); Perocchia et al. (2010).

Although the position paper by the NCI (2015) recommends the SPIKES protocol as a communication skills training tool for providers when delivering bad news to oncology patients, little research has specifically examined NPs’ communication with oncology patients. Since research supports that communication training improves the confidence of participants (Fujimori et al., 2014; Grainger et al., 2010; Mishelmovich, Arber, & Odelius, 2016; Skye et al., 2014) and the self-efficacy of health-care professionals from several disciplines (Bylund et al., 2011; Kissane et al., 2012), further research is needed specifically examining NPs and their experience with use of the SPIKES framework. To the best of the authors’ knowledge, research specifically with NPs in clinical practice has not yet been conducted in how the implementation of the SPIKES framework impacts them when delivering bad news.

**NCI’s Framework for Patient-Centered Communication in Cancer Care**

Improvement in the delivery of patient-centered communication has been identified as a priority in the NCI’s plan for research to reduce the pain and suffering of cancer patients. To deal with these issues, the NCI developed a conceptual framework built on six core functions to support both the provider and patient as a communication template for patient-centered cancer care (Epstein & Street, 2007; Perocchia et al., 2011). These functions include the following: fostering healing relationships, exchanging information, responding to emotions, managing uncertainty, making decisions, and enabling patient self-management. In the context of this study, these six elements served as a framework to support facilitation of individual interviews with participants and utilization of a communications skills’ training protocol for NPs when dealing with oncology patients.

## METHODS

**Design and Procedures**

After institutional review board approval was obtained, a flier announcing the research study was distributed to two professional groups: the Central Florida Chapter of the Oncology Nursing Society (CFONS) and the Greater Orlando Hematology Oncology Physician Extenders (GO HOPE).

Five NPs volunteered to participate in this two-part study utilizing a qualitative exploratory descriptive. In step one, a meeting with each NP was conducted to obtain informed consent and provide education on the use of the SPIKES Six-Step Protocol for Delivering Bad News to Patients (Table 1). Participants were asked to employ the tool in their clinical practice for 30 calendar days (1 month).

Thirty days later in step two, individual one-on-one interviews were conducted and recorded. Interview questions consisted of five scripted, open-ended, qualitative discussion questions, which were developed based on the NCI’s Conceptual Framework for patient-centered communication in cancer care, and are listed here (Figure; Epstein & Street, 2007; Perocchia et al., 2010).

Nurse practitioners were asked to share their perceptions, experiences, and insights on the changes or differences the protocol made in their relationships with patients. Additionally, participants were questioned about the tool’s usefulness in helping patients in problem-solving and decision-making. To maintain patient privacy, the researcher discouraged all discussion regarding specific patients, and no data were collected on any specific patient.

**Sample**

Participants were female, with an average age of 33.8 years, and had 2 to 10 years of professional experience. All worked in private practice, with three reporting associated acute care duties. Two participants had Doctorate of Nursing Practice degrees (DNP) and three had Masters of Science in Nursing degrees (MSN). Three participants were certified as "Advanced Oncology Certified Nurse Practitioners," and two were certified as "Adult Nurse Practitioners."

**Data Analysis**

Interviews were transcribed by the investigator, and a six-step process conducting thematic network analysis was utilized to extract common themes from individual interviews; they were used to evaluate and understand each study objective (See Table 2; Terry, 2012). Validity and reliability were achieved by employing triangulation, saturation, concept checking, and bracketing.

**Table 2:**
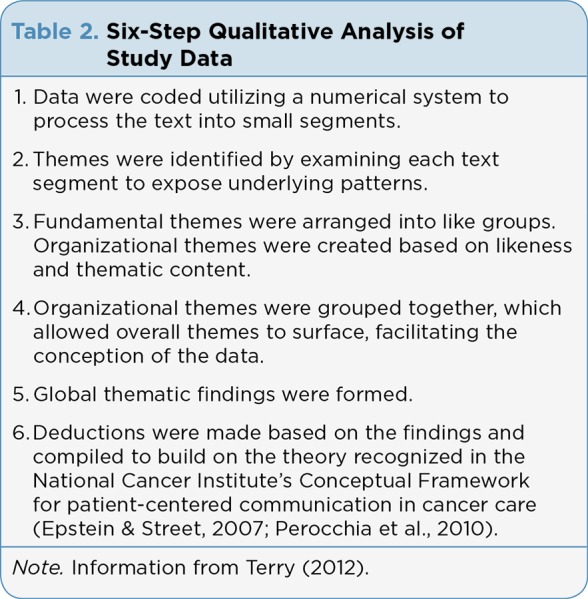
Six-Step Qualitative Analysis of Study Data

**Results and Discussion of Themes**

As part of the individual interviews, participants were asked five questions regarding their use of the SPIKES protocol when delivering bad news to cancer patients in practice.

***Question 1: Did the SPIKES protocol foster a healing, compassionate relationship between you and your patient when bad news was communicated?***

Themes that arose from participants’ responses supported the concept that the SPIKES protocol helped to establish a relationship between patient and NP by providing a supportive relationship. Subthemes that arose included (1) being deliberate; (2) developing a sense of compassion; (3) becoming more personable; (4) limiting misunderstanding; and (5) giving it time.

***Question 2: Did the SPIKES protocol help you to respond to the emotions exhibited by your patients in a sensitive manner, allowing for their cultural and spiritual beliefs?***

Themes that arose from participants’ responses supported the concept that the SPIKES protocol builds understanding that promotes emotional support. Participant subthemes related to this question included promoting emotional support through (1) understanding; (2) watching emotions; (3) recognizing and respecting; and (4) creating space consideration.

***Question 3: Did the SPIKES protocol help to support your patients in problem-solving and encourage them to participate in decision-making related to their care?***

Themes that arose from participants’ responses supported the concept that the SPIKES protocol supports patient understanding, leading to patient problem-solving and decision-making. Subthemes related to this question included (1) patient problem-solving; (2) patient decision-making; and (3) patient understanding.

***Question 4: What are the facilitators provided by the SPIKES protocol when trying to create a healing environment for your patients that respects their human dignity when giving them bad news?***

Themes that arose from participants’ responses supported the concept that the SPIKES protocol promotes a positive environment when delivering bad news. Subthemes that arose included (1) promoting a quiet environment; (2) creating a private environment; and (3) intentionally arranging the best setting for difficult conversations.

An additional theme that arose from participants’ responses to this question supported the concept that the SPIKES protocol supports a respectful environment that provides human dignity, and subthemes included (1) not rushing; (2) respect; and (3) intentionally supportive body language.

***Question 5: What elements of the SPIKES protocol helped you to promote patients’ self-management while meeting their basic physical, emotional, and spiritual needs?***

Themes that arose from participants’ responses supported the concept that the SPIKES protocol allows for information giving that supports self-management and decision-making. Subthemes included (1) understanding; (2) self-management; (3) decision-making; (4) partnering; and (5) giving information.

A second concept that emerged from participants’ responses to this question was the concept that the SPIKES protocol promotes holistic patient care. Subthemes that arose from participants’ responses included (1) physical; (2) emotional; (3) spiritual; and (4) cultural.

Finally, results from this study revealed there were common global themes among participants when utilizing the SPIKES protocol. Utilizing Terry’s (2012) thematic analysis process, in four of the five interview questions, all participants shared common themes. They included seven global themes obtained from the data. These themes include that the SPIKES protocol:

- Helps to establish a relationship between patients and NPs
- Builds understanding that promotes emotional support
- Supports patient understanding, leading to patient problem-solving and decision-making
- Promotes a positive environment when delivering bad news
- Supports a respectful environment that promotes human dignity
- Allows for information giving that supports self-management and decision-making
- Promotes holistic patient care.

Based on the thematic analysis of the study objectives and their supporting global themes, results support the overarching concept that "the experiences of the nurse practitioner when delivering bad news to cancer patients are shaped by their own communication skills."

## DISCUSSION

This type of qualitative research is of great value in generating an understanding of how utilizing the SPIKES protocol positively impacts how NPs deliver bad news to patients. The resulting themes suggest this tool is as useful for MDs as it is for NPs. These findings support that communication training and using a standardized tool play an important part in improving not only patient experiences but also provider experiences. Although PAs were not included in this research sample, it is reasonable to conclude they too would benefit from additional communication training and use of a protocol to help them compassionately deliver bad news.

Communication training should be included in all providers’ educational programs and often is now standard; however, since providers shape their own experiences, this research implies that repeat education and training in communication (beyond initial introduction of the concept in prelicensure and graduate programs) may be necessary to hone providers’ skills and facilitate holistic patient care. Limitations of this study include the following: (1) its small sample size; (2) participants consisted of only female NPs; (3) it is from one geographic area; (4) it focused on private practice; (5) the ages of the participants is similar; thus, the findings may not be generalizable to other practitioners, genders, and practice settings. Additionally, there may be a bias, as participants may have wanted to please the researchers. Future research should replicate this study with physician assistants as well as represent a more diverse mix of gender, ages, and geographic areas. Examining whether the use of this tool improved confidence for all health-care providers, the relationship of provider burnout, and occupational commitment to communication skills training are other areas of opportunity for new research.

## CONCLUSION

Delivering bad news is an unavoidable part of an oncology practitioner’s role. Promoting emotional support of patients while improving the experience for practitioners is essential. As self-reported, all five participants indicated they would continue to use the protocol, teach its methods to their peers in practice, and recommend it to others.
